# Laser Powder-Bed Fusion as an Alloy Development Tool: Parameter Selection for In-Situ Alloying Using Elemental Powders

**DOI:** 10.3390/ma13183922

**Published:** 2020-09-04

**Authors:** Leonardo Shoji Aota, Priyanshu Bajaj, Hugo Ricardo Zschommler Sandim, Eric Aimé Jägle

**Affiliations:** 1 Lorena School of Engineering, University of São Paulo, Lorena SP 12602-810, Brazil; l.aota@mpie.de (L.S.A.); hsandim@demar.eel.usp.br (H.R.Z.S.); 2 Department Microstructure Physics and Alloy Design, Max-Planck-Institut für Eisenforschung GmbH, 40237 Düsseldorf, Germany; eric.jaegle@unibw.de; 3 Institute of Materials Science, Universität der Bundeswehr München, 85579 Neubiberg, Germany

**Keywords:** laser powder-bed fusion, Additive Manufacturing, in-situ alloying, alloy development

## Abstract

The design of advanced alloys specifically tailored to additive manufacturing processes is a research field that is attracting ever-increasing attention. Laser powder-bed fusion (LPBF) commonly uses pre-alloyed, fine powders (diameter usually 15–45 µm) to produce fully dense metallic parts. The availability of such fine, pre-alloyed powders reduces the iteration speed of alloy development for LPBF and renders it quite costly. Here, we overcome these drawbacks by performing in-situ alloying in LPBF starting with pure elemental powder mixtures avoiding the use of costly pre-alloyed powders. Pure iron, chromium, and nickel powder mixtures were used to perform in-situ alloying to manufacture 304 L stainless steel cube-shaped samples. Process parameters including scanning speed, laser power, beam diameter, and layer thickness were varied aiming at obtaining a chemically homogeneous alloy. The scientific questions focused on in this work are: which process parameters are required for producing such samples (in part already known in the state of the art), and why are these parameters conducive to homogeneity? Analytical modelling of the melt pool geometry and temperature field suggests that the residence time in the liquid state is the most important parameter controlling the chemical homogeneity of the parts. Results show that in-situ alloying can be successfully employed to enable faster and cost-efficient rapid alloy development.

## 1. Introduction

Additive manufacturing (AM), unlike conventional processing methods, is based on the addition and consolidation of small portions of powder feedstock, layer by layer, aimed at producing dense parts with complex shapes [1,2]. The part is obtained with the aid of a computer-aided design (CAD) model, which is sliced in virtual layers in preparation for the AM process.

Laser Powder Bed Fusion (LPBF), also termed Selective Laser Melting (SLM), is an AM process based on the melting of a thin powder layer [1] under inert gas atmosphere (at atmospheric or reduced pressure) by scanning a high-power laser beam across the powder bed in a pre-defined scanning strategy. [1,3]. Cooling rates as high as 10^6^ K/s [4] are estimated due to the small melt pools and the high laser scanning speeds [5], resulting in fine and anisotropic microstructures, typically dominated by solidification structures such as cells or dendrites. It should be noted that the behavior of the liquid metal in the melt pool is influenced by several concurrent physical phenomena, such as Marangoni convection [6,7,8,9] and recoil pressure from the metal vapor above the melt pool [6,7] generated by the laser.

The quality of the starting powder batch plays an important role in determining the soundness of the final part. Powder chemistry, surface roughness, and particle size distribution affect how the laser radiation is absorbed, and consequently how deep the melt pool becomes [10,11,12]. Spherical particles with a smooth surface and Gaussian size distribution are preferred for better powder flowability, resulting in a uniform and more packed powder beds [1,13].

The powders usually chosen for LPBF processing are pre-alloyed, i.e., already contain all chemical elements required in the final part and have a fine size fraction of 15–45 or 20–60 µm [13]. The gas atomization processes that are mostly used today to produce spherical particles with smooth surfaces, both free-fall and close-coupled gas atomization, typically produce powder size distributions for which only half or less of the powder mass obtained falls within the size range required for LPBF processing [14]. This makes working with small powder batches for prototyping or alloy design purposes rather expensive.

A more time- and cost-efficient way to develop new alloys for AM is to completely avoid the use of pre-alloyed powders. Instead, it is preferable to use mixtures of elemental powders that homogenize in the melt pool (in-situ alloying). Such in-situ alloying is a well-established approach for directed energy deposition (DED), a process in which powder is transported into the melt pool, generated by a laser beam, via an inert carrier gas (see e.g., [15,16]). The laser powers typically used in DED are an order of magnitude larger than in LPBF, resulting in a melt pool whose width and depth is also typically an order of magnitude larger (100–200 µm for DED [17] compared to 50–300 µm for LPBF [18]). Due to the greater layer heights and slower scan speeds, the energy densities (defined as the ratio of the laser power to the product of scan speed, layer height, and beam diameter) used in DED and LPBF are often quite similar. The available geometrical complexity, spatial resolution, and cooling rates of DED, however, are much lower than in LPBF, leading to much higher popularity of the latter process in industrial applications. Metal matrix composites have also been processed by mixing different powders in DED [19,20,21] and LPBF [22,23].

In-situ alloying with the aim of achieving chemical homogeneity during LPBF processing has also been widely studied. Several works have shown that samples with relative densities >98% can be manufactured using mixtures of elemental powders and in-situ alloying during LPBF processing [24,25,26,27,28,29,30,31,32,33]. However, chemical inhomogeneity in the as-produced samples was reported by the researchers for many different materials including Ti alloys [24,25,29], Al alloys [30,31,33], high entropy alloys [27], and austenitic steels [32]. Grigoriev et al. [24] reported unmelted and partially melted Al and Nb particles in the as-produced samples. They were able to achieve chemical homogeneity after isothermal annealing at 1250 °C for 2.5 h. Similarly, unmelted and partially melted Si particles were observed for in-situ alloying of Al-Si alloys [30,31]. Kang et al. [31] studied the effects of process parameters on Si agglomeration. Their results show that slower scan velocities promote elemental mixing during LPBF processing. Similarly, Strauss et al. [32] reported that the use of higher laser power improved chemical homogeneity in in-situ alloyed Invar36 samples produced using elemental Fe and Ni powders in LPBF. They were able to achieve chemical homogeneity after isothermal annealing at 1310 °C for 90 min. Karg et al. [33] also report an improvement in chemical homogeneity after heat treatment in their in-situ alloyed aluminum alloy. Dry coating Al powder particles with SiOx nanoparticles allowed them to use a larger fraction of the powder size fraction with particles smaller than 20 µm also being usable due to reduced interparticle attraction which results in poor flowability.

The effect of volumetric energy density on the relative densities of the samples produced by in- situ alloying has also been studied [28]. High energy densities result in keyhole pores in the sample, while low energy densities lead to lack of fusion porosity. The highest relative density was obtained using intermediate energy densities. Mixing pure Fe powder with pre-alloyed SS 304 powders resulted in relatively homogeneous samples [34]. Dzogbewu et al. [35] studied in-situ alloying of Ti-15Mo alloy using elemental Ti and Mo powders in LPBF. Their results show that laser power and velocity have a strong effect on the stability of single tracks, with the range of scanning speeds to produce stable tracks increasing with increasing laser power. They also showed that re- melting improved the chemical homogeneity of the in-situ alloyed samples [35]. In none of these studies, was it clarified via which mechanism the varied process parameters led to the resulting chemical homogeneity or inhomogeneity.

Here, we use austenitic stainless steels as model alloys for demonstration of the in-situ alloying technique in LPBF processing. These alloys are routinely produced by LPBF with high relative density (>99%) [18,36,37,38,39]. Often, the achieved relative density is related to the volumetric energy density ($E_{o}$) employed in the process, a parameter that is easy to calculate, but limited in its predictive power [40,41]. Bajaj et al. [41] proposed a more predictive approach to process parameter optimization using analytical heat transfer models.

In this study, we investigated how the development of new alloys for LPBF, i.e., the capabilities of LPBF as rapid alloy prototyping technique can be improved. We performed in-situ alloying using elemental powders to build AISI 304 L steel parts via LPBF and analyzed which process parameters lead to a chemically homogeneous part. For this analysis, we extended the predictive approach by Bajaj et al. [41] for application to stainless steels and calculated additional features of the melt pool, such as residence time and freezing rate. We not only varied the laser power and scan speed but also the laser beam diameter and hence the melt pool diameter. We aimed to show that in-situ alloying in LPBF can be successfully employed by choosing suitable process parameters, enabling faster and less cost-intensive alloy development in the future. We also attempted to find which process parameters are required for producing such samples (partially already known in state of the art), and why are these parameters conducive to homogeneity.

## 2. Materials and Methods

### 2.1. LPBF Experiments

**Figure A1:**
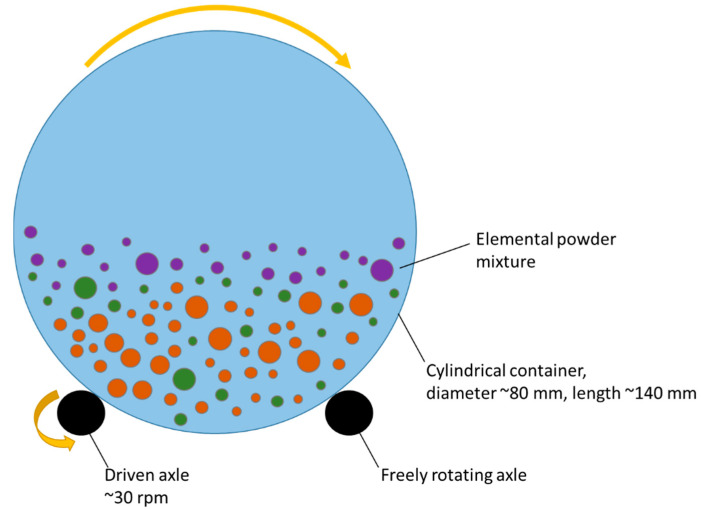
Schematic representation of the roller mixer used for mixing of elemental powder.

Pure elemental iron, chromium, and nickel gas atomized powders (TLS Technik GmbH & Co. Spezialpulver KG, Bitterfeld, Germany) in the size range 20–53 µm were thoroughly mixed in a roller mixer (see Figure A1 in the Appendix A) for durations from 5 to 20 h in the proportion Fe-18Cr-14Ni (wt %). The mixed powder was processed by LPBF, to achieve a chemical composition similar to AISI 304 L stainless steel after processing, considering the eventual mass loss for Ni.

The samples were processed using an Aconity Mini LPBF machine (Aconity3D GmbH, Herzogenrath, Germany) equipped with a 400 W Nd laser source (λ = 1070 nm). The laser beam provides a Gaussian energy distribution on the surface. The machine can change the beam diameter from part to part using a “3D-scanner” (VarioScan by Scanlab GmbH, Puchheim, Germany). Then 10 mm-edge cube samples were built directly on a 316 L stainless steel substrate plate, without heating, under an argon atmosphere with an oxygen content below 100 ppm. A cross-hatching pattern (90° rotation between the layers) scanning strategy was used. For each set, a single cube was processed and all data were obtained from this sample. For some samples, re-melting was applied, which consists of melting the same surface twice using the same parameters, before a new powder layer is added. We used a modified form of volumetric energy density ($E_{o}$) defined as follows (Equation (1)):

(1) $$E_{o}=\frac{Q}{2\;v\;r_{b}\;l}$$

where Q is the laser power, v is the scanning speed, l is the layer thickness, and $r_{b}$ is the beam radius. A more commonly used form of volumetric energy density ($E_{o}^{h}$) is based on hatch spacing instead of the laser beam radius, which is normally kept constant. This is defined as following (Equation (2)):

(2) $$E_{o}^{h}=\frac{Q}{v\;h\;l}$$

Different process parameters used for each sample are summarized in Table 1. The goal of the parameter selection was to scan over a broad range of laser powers, layer thickness, beam diameter, and scan speeds while keeping the energy density (nearly) constant. In this case we did not perform a full factorial DOE.

### 2.2. Characterization Techniques

Standard metallographic preparation was used to reveal the microstructure. Samples were ground using #800–#1200 SiC abrasive paper, followed by diamond polishing using a 3 µm diamond suspension. The finishing step was a polishing using a 40 nm colloidal silica suspension. Sample cleaning using isopropanol in ultrasound was applied between each preparation step. Porosity was determined by applying the threshold function of the ImageJ software (version number 1.52r, NIH, Bethesda, Rockville, MD, USA) on light optical microscopy images obtained from a single cross-section. The entire cross-section (10 × 10 mm^2^) was captured, resulting in around 5–7 (50× magnification) images per sample. Average and standard deviation were obtained based on these images. The selection of this method for porosity measurement was based on an earlier work by Spierings et al. [42]. Scanning electron microscopy was performed in a Zeiss Merlin FEG-SEM (Carl Zeiss Microscopy GmbH, Jena, Germany) and also in a JEOL-6500F microscope (JEOL (Germany) GmbH, Freising, Germany).

Electron backscatter diffraction (EBSD) maps (300 × 300 µm^2^) with 500 nm step size were obtained in the JEOL-6500F scanning electron microscope with an acceleration voltage of 15 kV and a TSL TexSEM DigiView EBSD camera (EDAX, Mahwah, NJ, USA). A standard data clean-up procedure was adopted to remove points with confidence index lower than 0.1. Energy dispersive X-ray spectroscopy (EDS) was performed in the same microscope along a line of 1 mm length, divided into 200 points using an EDAX Octane Plus EDS detector with a dwell time of 2 s. EDS maps (500 × 400 µm^2^) were also collected for 30 min using a 200 μs dwell time. EDS measurements only considered the main alloy elements (Fe, Cr, and Ni). All microstructural characterizations were performed on a surface parallel to the building direction (BD). SEM, using the secondary electrons mode, and EDS were also performed for the initial powders.

### 2.3. Analytical Melt Pool Modelling

We used the modification of Ashby et al. [43] of the Rosenthal solution [44] for a moving heat source with finite size to model the melt pool geometry. It describes the time-dependent temperature at a point, z distance, directly below the center of the beam as it travels. This approach is described in detail in an earlier paper using the original Rosenthal equation for application to high conductivity metals [41]. Note that this simplistic analytical solution is not an accurate description of the melt pool as it does not account for parameters such as latent heat. However, since the deviations because of these exclusions are systematic, it can still be used for a comparative study. The temperature field T(z,t) is described as:

(3) $$T\left(z,t\right)=T_{o}+\frac{nQ}{2\pi k_{t}v{\left[t\left(t+t_{o}\right)\right]}^{0.5}}\;\exp-\left(\frac{{\left(z+z_{o}\right)}^{2}}{4\alpha t}\right)$$

where t=0 when the laser is directly above the point. n is laser absorptivity, α is thermal diffusivity, and $k_{t}$ is thermal conductivity. $t_{o}$ and $z_{o}$ are defined as follows:

(4) $$t_{o}=\frac{r_{b}{}^{2}}{4\alpha }$$

(5) $$z_{o}{}^{2}=\sqrt{\frac{\pi \alpha r_{b}}{v}}\;\frac{r_{b}}{\exp\left(1\right)}$$

## 3. Results

### 3.1. Powder Morphology and Chemistry

**Figure 1:**
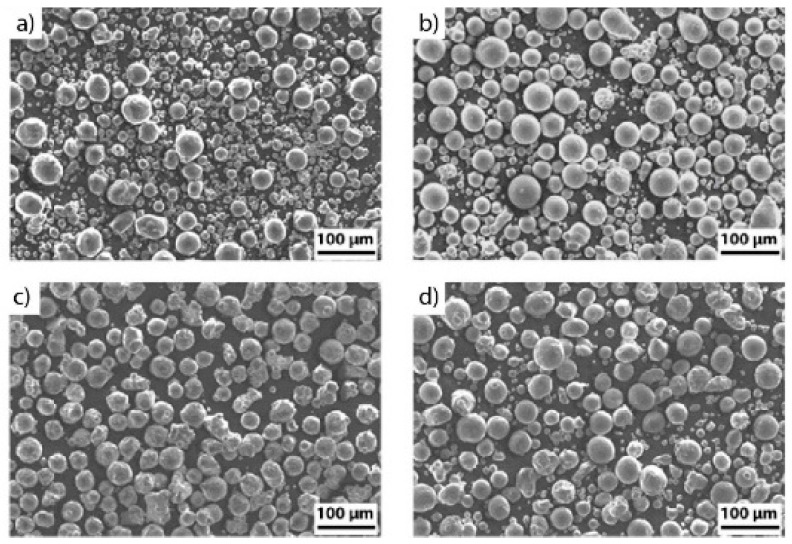
SEM micrographs depicting Fe (**a**), Cr (**b**), Ni (**c**) elemental powders, and mixture of Fe, Cr, and Ni powders (**d**).

Elemental Fe, Cr, and Ni powders are shown in Figure 1a–c, respectively. Fe and Ni powders have satellite particles, i.e., small particles attached to the surface of coarser particles, as well as some irregular particles. These defects are inherited from the atomization process [3]. Chromium particles are spherical and no significant surface defects can be noticed. The mixture of the elemental powders appears to be homogeneous as depicted in Figure 1d. The chemical composition of the elemental iron, chromium, and nickel powders, as well as the standard AISI 304 L composition given by ASTM A240, and LPBF-produced in part after in-situ mixing (obtained via ICP-OES—Inductively coupled plasma—optical emission spectrometry) are given in Table 2. ICP-OES measurement was performed on the sample with the following process parameters: Q = 380 W, v = 0.3 m/s, h = 120 μm, l = 70 μm, and $r_{b}$ = 100 μm, due to its higher chemical homogeneity.

### 3.2. Relative Density

**Figure 2:**
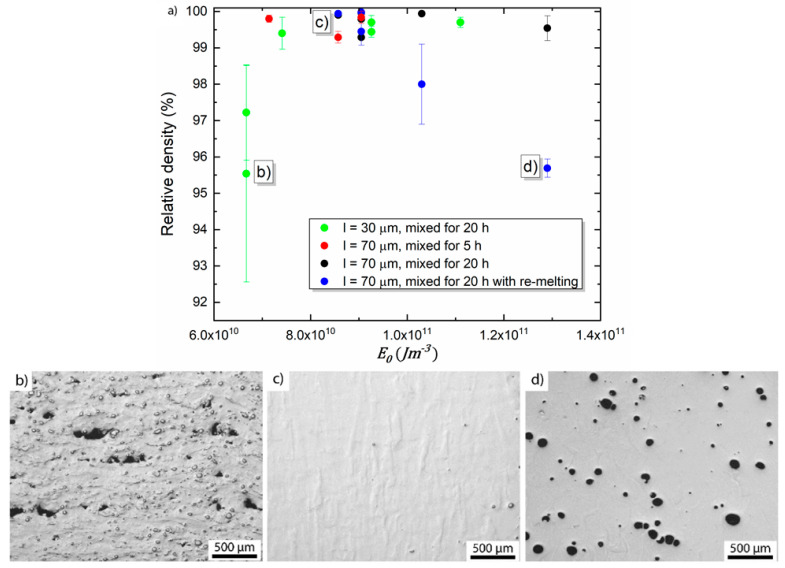
(**a**) Relative density as a function of $E_{o}$. In (**b**), lack of fusion defects as well as unmelted powder particles are found for low $E_{o}$. Sample parameters are Q = 360 W, v = 2 m/s, h = 80 μm, l = 30 μm, and $r_{b}$ = 45 μm. (**c**) Full density for intermediate values of $E_{o}$. Sample parameters are Q = 360 W, v = 0.3 m/s, h = 120 μm, l = 70 μm, and $r_{b}$ = 100 μm. (**d**) Keyhole defects are observed for high $E_{o}$ and re-melting. Samples parameters are Q = 380 W, v = 0.3 m/s, h = 120 μm, l = 70 μm, and $r_{b}$ = 100 μm. Building direction is upward for all images.

In Figure 2a the relative densities of all samples produced by mixing elemental (pure) powders are shown (cf. also Table 1) as a function of the volumetric energy density ($E_{o}$). Data were obtained from quantitative metallography and error bars represent the standard deviation of all individual measurements (micrographs) per sample. This includes the samples produced with two different powder mixing times, as well as with two different layer thicknesses, 30 and 70 µm.

As expected, the relative density drops both at low and high energy densities. High relative densities (>99%) were observed at intermediate $E_{o}$ values, albeit, with some scatter. Increasing the mixing time from 5 to 20 h did not impact the relative density. Since in our experiments we compensate for an increase in layer thickness with an increase in laser power and beam diameter, generating larger melt pools, the variation in layer thickness, by itself, has no systematic effect on the relative density. For samples processed with low energy density, defects such as lack of bonding and unmelted particles were observed (cf. Figure 2b). Re-scanning of each layer leads to the elimination of these defects (cf. Figure 2c), and hence to an increase in relative density. Re-scanning also leads to a higher total input of thermal energy, and therefore, to heat accumulation in the sample being built. This overheating shifts the onset of keyhole formation (see Figure 2d) to lower $E_{o}$ values (9.0 × 10^10^ J/m^3^) vis a vis non-remelted samples, where we did not see the onset of keyhole formation within the range of process parameters used in this work (see Figure 2a). This occurs since the $E_{o}$ value does not include the effect of re-melting [40].

### 3.3. Chemical Inhomogeneity in As-Produced Samples

**Figure 3:**
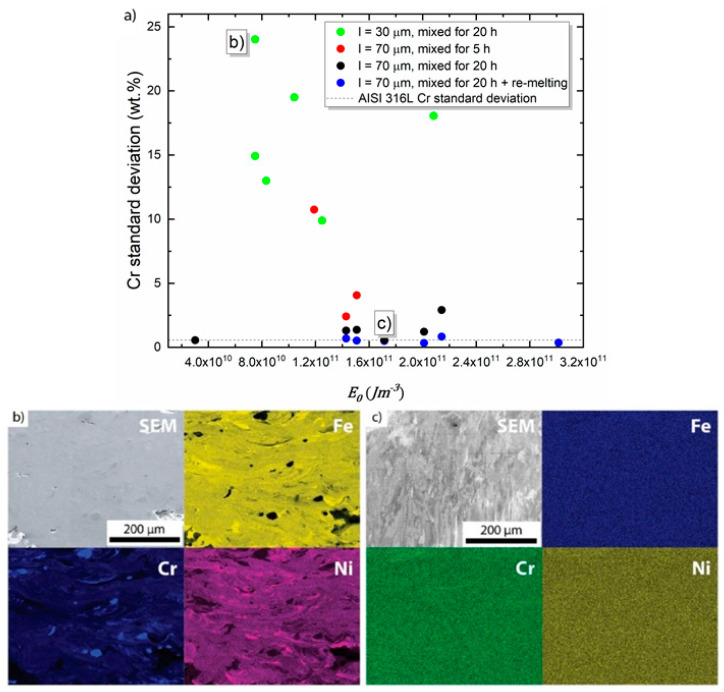
(**a**) Standard deviation of the Cr content from different process parameters as a function of $E_{o}$. EDS elemental maps from the samples from Figure 2b (**b**) and Figure 2c (**c**). High Ni and Fe inhomogeneity, and unmelted Cr particles are observed for the low $E_{o}$ sample (b). Sample parameters for (b) are Q = 360 W, v = 2 m/s, h = 80 μm, l = 30 μm, and $r_{b}$ = 45 μm. Full chemical homogeneity at this length scale was observed for intermediate $E_{o}$ and re-melting scan sample (c). Sample parameters for (c) are Q = 360 W, v = 0.25 m/s, h = 120 μm, l = 70 μm, and $r_{b}$ = 100 μm. Building direction is upward for all images.

EDS analysis of the sample shown in Figure 2b shows highly heterogeneous distributions of Fe, Cr, and Ni (see elemental maps in Figure 3b). Only these elements were tracked, since they are the main elements used to check if we achieved our desired (AISI 304 L similar) chemical composition. Note that no Mn addition was made to our alloy and hence it was not measured. The chromium particles are insufficiently melted and remain as dispersed spherical particles (cf. Figure 2b and Figure 3b). It appears that the Fe and Ni particles did melt completely, however, the mixing in the melt pool was still insufficient. The concentration inhomogeneity for Fe and Ni roughly follows the melt pool shape. Since Cr was the most heterogeneously distributed element in Figure 3b, we selected Cr inhomogeneity as a tracer to quantify the degree of chemical homogeneity within the samples. The standard deviation of 200 measurement points on a 1-mm long EDS line scan arbitrarily placed on the samples’ surfaces, along the build direction, was calculated. Note that this line spans over many layer thicknesses and includes many unmelted powder particles, if present.

**Figure A2:**
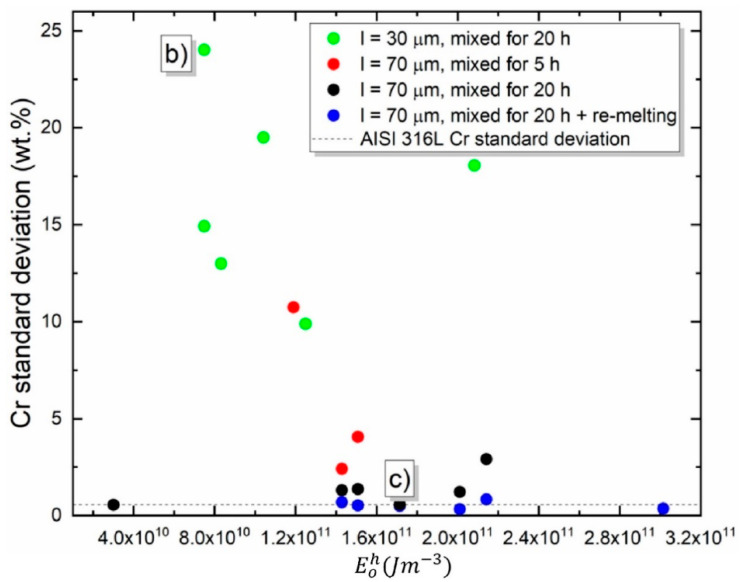
Standard deviation of the Cr content from different process parameters as a function of $E_{o}^{h}$, the volumetric energy density calculated based on the hatch spacing.

Figure 3a and Figure A2 (in the Appendix A), shows the relationship between the standard deviation of the Cr content and $E_{o}$ and $E_{o}^{h}$ respectively. In general, higher $E_{o}$ values yield more chemically homogeneous samples. Raising the powder mixing time before LPBF processing from 5 to 20 h enhances powder mixing and the corresponding LPBF-produced samples are chemically more homogeneous. Powders, therefore, were mixed for all subsequent experiments for 20 h. The 30 µm layer thickness samples were chemically more heterogeneous than the samples processed with a 70 µm layer thickness. All samples with re-melting scans show a Cr standard deviation of less than 1%, close to the scatter observed in a commercial AISI 316 L (recrystallized) sample. Our results are significantly better than earlier studies on in-situ alloying of a similar alloy by Strauss et al. [32] who reported a minimum standard deviation in chemical composition of 5.3% in the as-produced state. The AISI 316 L is used for comparison since we assume the chemical homogeneity must be similar between Fe-Cr-Ni stainless steel in the annealed condition. Figure 3c shows EDS maps for a chemically homogeneous in-situ alloyed AISI 304 L sample (shown in Figure 2c).

**Figure 4:**
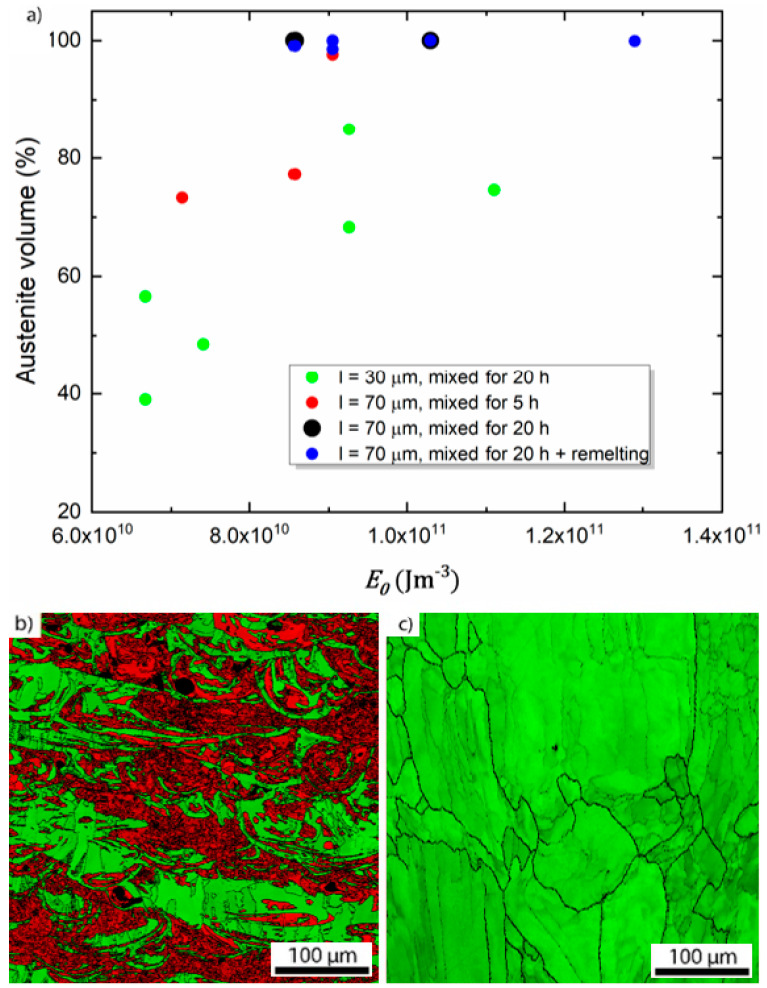
(**a**) Austenite volume fraction as a function of $E_{o}$ obtained from EBSD measurements. EBSD map for sample parameters Q = 300 W, v = 1.5 m/s, h = 80 μm, l = 30 μm, and $r_{b}$ = 45 μm (**b**). (red: ferrite, green: austenite). EBSD map for sample parameters Q = 360 W, v = 0.25 m/s, h = 120 μm, l = 70 μm, $r_{b}$ = 100 μm and re-melting (**c**). (fully austenitic). Building direction is upward for all images.

Another homogeneity indicator is the phase volume fraction. Conventionally-produced AISI 304 L steel is fully austenitic (A1/Cu-type crystal structure). Also, material produced by DED only contains small amounts of residual δ-ferrite (A2, W-type) [45]. However, any inhomogeneity of ferrite-stabilizing (Cr) and austenite-stabilizing (Ni) elements can be expected to result in the presence of two phases in the microstructure. In Figure 4a, austenite volume fractions as determined by EBSD scans are plotted against $E_{o}$ for different process parameters. Note that the EBSD measurements at a relatively coarse step size of 500 nm could miss very small regions of residual (δ) ferrite. This has been reported in the literature for DED-produced [46,47], but not for LPBF-produced austenitic stainless steels [48,49,50,51,52].

Again, a higher energy density leads to an increase in the chemical homogeneity which, in turn, raises the austenite fraction. All samples produced with a layer thickness of 30 µm result in a considerable ferrite (A2) phase fraction. In other words, chemically heterogeneous and not fully austenitic material is produced even for the highest $E_{o}$ used when employing a smaller layer thickness. An example of such a sample produced with a 30 µm layer thickness sample is shown in Figure 4b where the volume fraction of ferrite is about 51.5%. Phases are distributed following the melt pool shape, similar to the concentration inhomogeneity observed in Figure 3b. Cr-rich areas cannot be clearly distinguished by EBSD since their crystallographic structure and lattice parameter are similar to the ferrite. Therefore, the Cr-rich areas were accounted as ferrite.

Increasing both layer thickness (and at the same time the beam diameter) and mixing time leads to better chemical homogeneity, and therefore, to a higher austenite volume fraction. For the highest energy density values, ferrite is reduced to just a few percent. Finally, re-melting every layer leads to fully austenitic samples for all process parameters, as seen in the example depicted in Figure 4c. The targeted chemical composition was reached in these samples with satisfactory accuracy. The overall composition as measured by ICP-OES is Fe-17.1Cr-11.8Ni (wt %, cf. also Table 2).

## 4. Discussion

Full chemical homogeneity was achieved in our experiments using mixtures of elemental powders. In the first set of experiments, a thorough mixing and complete homogenization of the powder blend was shown to be a prerequisite to obtaining homogeneous samples after LPBF. This feature highlights the first limitation of this method of alloy synthesis; i.e., mixtures of elements of very different densities are likely to remain inhomogeneous after powder blending or to re-segregate quickly and hence the final samples might not have the targeted composition and homogeneity. Also, if one of the elemental powders shows an irregular surface or morphology, its poor flowability may lead to heterogeneous spreading [13]. The poor flowability and density difference between elemental powders may be overcome by using finer particles (<20 μm) coated with SiOx nanoparticles, which act as spacers and reduce the attraction between powder particles [33,53]. Note that the simple rolling mixer used in this study is not an ideal way to produce homogeneous powder blends, so better mixers are expected to achieve a higher homogeneity in a much shorter time. It should be highlighted that we used in-situ alloying with a focus on facilitating alloy development where large samples are normally not required, i.e., testing new alloy compositions. For example, the processability and properties of a new alloy composition may be tested under laser powder-bed fusion conditions by in-situ alloying. By employing this method, the composition may be tuned until the desired microstructure and properties are achieved. A pre-alloyed powder could be acquired for further large-scale testing and manufacturing. The use of in-situ alloying during the initial iterative process, instead of using pre-alloyed powders of multiple compositions, helps in significantly reducing the cost and time spent on alloy development studies.

Once the correct powder blending is ensured, it was noticed in the next sets of experiments that a large melt pool is beneficial to chemical homogenization. A higher energy density and a higher layer thickness (combined with a larger laser beam diameter) both lead to larger melt pools and hence a better homogenization of the melt. It is important to distinguish between a wide melt pool as a result of a large laser beam diameter and a narrow-and-deep melt pool as a result of keyhole formation. While the latter possibly also leads to higher chemical homogeneity, it leads to porosity, and is therefore not within the target parameter window. Further, re-melting each layer was very effective in decreasing inhomogeneity. It should be noted that re-melting is not only achieved by scanning the laser several times over each layer. Decreasing the layer thickness, while keeping the melt pool depth constant (i.e., by keeping the laser power and scan speed constant) and decreasing the hatch spacing, also increases the number of times the material gets melted. However, since in our experiments the scan speed was increased when reducing the layer thickness (thus keeping the energy density constant), the melt pool depth decreased. Therefore, an improvement in the homogeneity with reduced layer thickness was not noticed; rather the opposite.

The obtained overall composition shows a slight deficiency of Cr (17.1 wt % instead of the targeted value of 18 wt %). This could be due to preferential loss of Cr during the LPBF process (e.g., by particle ejection from the melt pool or its vicinity). In future experiments, compensation by over- alloying of Cr could be used to reach the target composition in the LPBF-produced parts. Despite not reaching the target composition, the value is very close to the desired composition and the allowable composition for 304 L stainless steel. Note that some steel grades denoted ‘304 L’ are permitted to only contain 17.5 wt % Cr (e.g., 1.4307, X2CrNi18-9).

The input energy density is a poor predictor for chemical homogeneity. Depending on other process parameters such as layer height and remelting, identical values of $E_{o}$ yield very different Cr concentration fluctuations (cf. Figure 3a). To analyze in more detail which process conditions are beneficial for chemical homogeneity, the temperature profile present in the material during LPBF was calculated using a modified-Rosenthal approximation for a moving heat source with finite size (cf. Equation (2)). Even though this model is a simple analytical solution of thermal conduction and does not include many of the details that will impact homogenization, most notably fluid flow in the melt pool, it enables us to estimate the melt pool shape beyond what can be measured in micrographs. In particular, the melt pool length is experimentally not easily accessible, but it can be approximated by the model. It depends on the input power, beam diameter, and scanning speed, as well as on material properties such as reflectivity and thermal conductivity.

**Figure 5:**
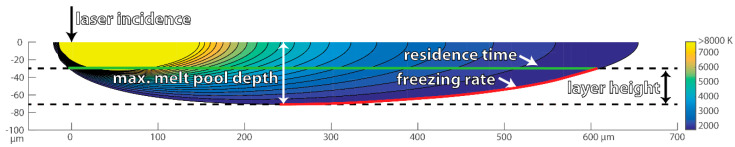
Schematic depiction of the simulated melt pool geometry (i.e., melting temperature isotherm). Some additional isotherms inside the melt pool are shown as well. The freezing rate is averaged over the region marked in red and the residence time is calculated using the length marked in green.

**Figure 6:**
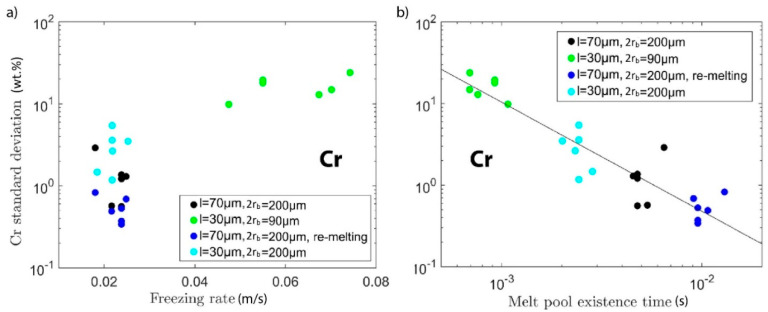
Re-plotting of the data from Figure 3, together with one additional set of experiments, as a function of simulated melt pool characteristics: as a function of average freezing rate of the melt (**a**) and as a function of the melt pool residence time, i.e., the time the material remains in the liquid state (**b**).

Using this modelling method, several hypotheses that could explain the impact of the processing conditions on measured compositional homogeneity were tested. The first hypothesis assumes that the freezing rate is the determining factor for homogeneity. A high solidification rate could lead to a “freezing in” of a chemically inhomogeneous melt pool. We approximate the freezing rate as the velocity of the melting temperature isotherm in the direction perpendicular to the isotherm. Taking into account that the top part of the melt pool will get remelted by the subsequent layer(s), the calculation was carried out by only averaging the freezing rates over the lower parts of the melt pool boundary; i.e., from the maximum depth of the melt pool up 30 or 70 µm, respectively (cf. the schematic in Figure 5). In Figure 6a, the measured Cr standard deviation, i.e., the same data as in Figure 3a, is plotted as a function of the thus obtained average freezing rate. Here we left out the data for experiments with incompletely mixed powder feedstock. While experiments with a small layer height and therefore a high freezing rate lead to a high chemical inhomogeneity, in general, there is no clear relationship between the homogeneity and the freezing rate and this hypothesis was dismissed.

**Figure 7:**
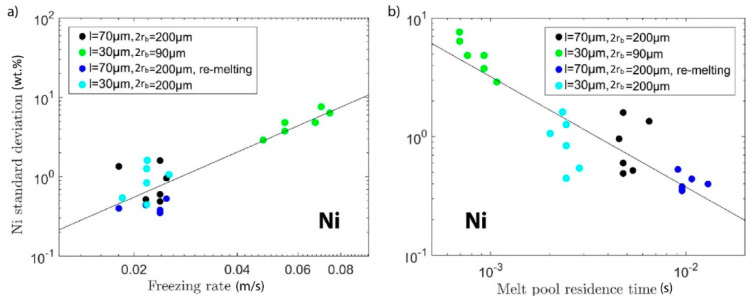
Same plots as in Figure 6, but with the analysis performed for Ni instead of Cr: the relationship between Ni standard deviation (wt %) and freezing rate (**a**) and melt pool residence time (**b**).

The next hypothesis is that a long time in which material is inside the melt pool, i.e., in the liquid state, is conducive to good mixing of the elemental powders. We call this the residence time and approximate it by dividing the maximum extent of the melt pool along the direction of laser travel by the laser scan speed. Only the length of the part of the melt pool that does not get re-melted in the next layer is taken into account, i.e., up from maximum depth by one-layer height (cf. schematic in Figure 5). In the case of re-melting, this number is multiplied by two. Again, the data of Figure 3a as a function of this calculated data were re-plotted. In the double-logarithmic plot of Figure 6b, a clear linear relationship between melt pool residence time and chemical homogeneity can be seen. This supports the hypothesis that long residence time in the liquid state is the most important factor for full mixing. The same analysis was performed for the Ni concentration fluctuation (see Figure 7), which yields the same conclusions as for the Cr concentration.

To test this hypothesis further, one more LPBF build with parameters carefully chosen to fill the gap in our data between residence times of 1 to 400 ms was performed. Testing various parameter combinations in the analytical model, we found that many of the parameter combinations that lead to residence times in the desired range require either excessive energy input likely leading to keyhole porosity, or very low energy input likely leading to high porosity. The chosen set of parameters (cf. Table 1) combines a rather large beam diameter (200 µm) with a low layer height (30 µm) and energy densities at the upper limit of the tolerable range established in Figure 2a. The resulting data points in Figure 6 and Figure 7 fit very well to the hypothesis that melt pool residence time is the main determining factor for chemical homogeneity.

The impact of melt pool residence time is qualitatively similar to the argument, presented above, that melt pool size is important for chemical homogeneity. Plots similar to Figure 6b can be constructed e.g., for the maximum melt pool depth and other melt pool properties following from size such as average cooling rates. However, none of these plots can take into account that re-melting improves homogeneity. Additional factors such as the velocity of the Marangoni convection [8] within the melt pool probably play an important role, too, in ensuring homogeneity and explain the data scatter. They were not included in the current simple analytical melt pool model.

The fact that chromium particles were not fully melted in some of the experiments with powder blends highlights another limitation of this technique. Only materials with relatively small differences in melting (and boiling) temperatures can be processed together. In the current experiments, the highest difference in the melting point was 452 K (Ni: 1728 K, Cr: 2180 K), and it was possible to fully melt the chromium particles and homogenize the melt by increasing the melt pool size. Melting of volatile elements may also present a limitation for composition control since these elements will be preferentially vaporized at higher temperatures [34]. In alloys including elements of higher melting temperature and vapor pressure difference, blending of several pre- alloyed master alloy powders could alleviate the problem while retaining the flexibility of the method. Another possibility is to use a smaller particle size for higher melting temperature materials while maintaining a coarser particle size for the remaining elements. A finer particle size requires less energy to melt due to a higher specific surface area [54]. The presence of fine particles in the powder- bed also leads to higher laser absorptivity due to multiple laser reflections, considering that they are in a significant amount [55,56].

Our study shows that using powder blends for alloy development in the LPBF process is a viable strategy. However, process parameters need to be carefully tuned to obtain chemical homogeneity. We argue that the most important factor to reach this homogeneity is a sufficiently long residence time of the melt pool. Thus, all processing parameter sets leading to large melt pools (and re-melting, if required) while keeping the energy density below the keyhole porosity threshold, should lead to the formation of a chemically homogeneous alloy directly after the LPBF process, without any post- process heat treatment.

## 5. Conclusions

1. Dense and chemically homogeneous AISI 304 L steel samples were obtained from in-situ alloying of elemental powders mixtures after process parameter optimization.
2. The comparison of our analytic, temperature-field based melt pool size modelling with experimental results (SEM-EDS) suggests that the most important factor to enhance the chemical homogeneity of LPBF-produced samples is the residence time in the liquid state. This can be achieved by parameter combinations leading to a large melt pool, or by repeated melting of material, or both.
3. Increasing both layer thickness (and at the same time the beam diameter) and mixing time leads to better chemical homogeneity, and therefore, to a higher austenite volume fraction.
4. Ensuring homogeneous powder mixture is also an important precondition to achieving chemical homogeneity.
5. Understanding which process parameters allow using elemental mixtures instead of pre-alloyed powders enables the use of the LPBF process in the future for rapid alloy development purposes without the need for homogenizing post-heat treatments.

## Figures and Tables

**Table 1:** Process parameters and relative densities for AISI 304 L steel by in-situ alloying during laser powder bed fusion (LPBF) processing. Here Q,v,l,h, and $r_{b}$ are defined as laser power, laser scan velocity, layer thickness, hatch spacing, and laser beam radius, respectively. $E_{o}\;$ and $E_{o}^{h}$ are volumetric energy densities defined as per Equation (1) and Equation (2), respectively.

Q (W)	v (m/s)	l (μm)	h (μm)	$2r_{b}$ (μm)	$E_{o}\;(J/m^{3})$	$E_{o}^{h}(J/m^{3})$
l **= 70 µm and mixed for 5 h**						
360	0.3	70	120	200	8.57 × 10^10^	1.43 × 10^11^
300	0.3	70	120	200	7.14 × 10^10^	1.19 × 10^11^
380	0.3	70	120	200	9.05 × 10^10^	1.51 × 10^11^
l **= 70 µm and mixed for 20 h**						
360	0.3	70	120	200	8.57 × 10^10^	1.43 × 10^11^
380	0.3	70	60	200	9.05 × 10^10^	3.02 × 10^10^
380	0.3	70	120	200	9.05 × 10^10^	1.51 × 10^11^
360	0.25	70	120	200	1.03 × 10^11^	1.71 × 10^11^
360	0.2	70	120	200	1.29 × 10^11^	2.14 × 10^11^
380	0.3	70	90	200	9.05 × 10^10^	2.01 × 10^11^
l **= 30 µm and mixed for 20 h**						
360	2	30	80	90	6.67 × 10^10^	7.50 × 10^10^
180	1	30	80	90	6.67 × 10^10^	7.50 × 10^10^
300	1.5	30	80	90	7.41 × 10^10^	8.33 × 10^10^
300	1	30	80	90	1.11 × 10^11^	1.25 × 10^11^
250	1	30	80	90	9.26 × 10^10^	1.04 × 10^11^
250	1	30	40	90	9.26 × 10^10^	2.08 × 10^11^
l **= 70 µm and mixed for 20 h with re-melting**						
360	0.3	70	120	200	8.57 × 10^10^	1.43 × 10^11^
380	0.3	70	60	200	9.05 × 10^10^	3.02 × 10^10^
380	0.3	70	120	200	9.05 × 10^10^	1.51 × 10^11^
360	0.25	70	120	200	1.03 × 10^11^	1.71 × 10^11^
360	0.2	70	120	200	1.29 × 10^11^	2.14 × 10^11^
380	0.3	70	90	200	9.05 × 10^10^	2.01 × 10^11^
**Further experiments** l **= 30 µm,** $2r_{b}$ **= 200 µm**						
200	0.3	30	120	200	1.11 × 10^11^	1.85 × 10^11^
200	0.25	30	120	200	1.33 × 10^11^	2.22 × 10^11^
200	0.2	30	120	200	1.67 × 10^11^	2.78 × 10^11^
300	0.3	30	120	200	1.67 × 10^11^	2.78 × 10^11^
300	0.25	30	120	200	2.00 × 10^11^	3.33 × 10^11^
300	0.2	30	120	200	2.50 × 10^11^	4.17 × 10^11^

**Table 2:** Chemical composition of elemental powders (as specified by the supplier) and LPBF- produced part after in-situ mixing (obtained via ICP-OES). Contents are expressed in wt %.

Sample (Bulk or Powder)	Fe	Cr	Ni	Mn	Si	C	Al
Fe powder	99.8	0.03	0.02	0.01	0.004	0.01	-
Cr powder	0.12	99.8	-	-	0.03	0.0037	0.01
Ni powder	<0.01	-	99.8	-	-	0.05	-
AISI 304 L (ASTM A240)	Bal.	18–20	8–12	<2	<0.75	<0.03	-
LPBF-produced part after in-situ mixing	Bal.	17.1	11.8	-	-	-	-
